# How Personality Traits Affect the Perception of Facial and Vocal Attractiveness

**DOI:** 10.3390/brainsci15111143

**Published:** 2025-10-25

**Authors:** Lingyun Xiang, Werner Sommer, Siqi Yue, Jingyu Liao, Meng Liu, Weijun Li

**Affiliations:** 1 Institute of Psychological and Brain Sciences, Liaoning Normal University, Dalian 116029, China; xiang182083@163.com (L.X.); ysiqi777@163.com (S.Y.); 2 Key Laboratory of Brain and Cognitive Neuroscience, Dalian 116029, China; 3 Department of Psychology, Humboldt-Universität zu Berlin, 10117 Berlin, Germany; werner.sommer@cms.hu-berlin.de; 4 Faculty of Education, National University of Malaysia, Kuala Lumpur 50300, Malaysia; 5 Department of Physics and Life Sciences Imaging Center, Hongkong Baptist University, Hong Kong 999077, China; 6 Department of Psychology, Liaoning Normal University, Dalian 116029, China; ljy04232022@163.com

**Keywords:** facial attractiveness, vocal attractiveness, personality trait words, ERP

## Abstract

Background: Previous research has found an association between attractiveness and personality traits, but the neural mechanisms are largely unknown. Method: We used a Stroop-like paradigm combined with EEG recordings to investigate how personality traits affect the perception of facial and vocal attractiveness. Twenty-three female participants classified the attractiveness of male faces and male voices paired with positive or negative personality trait words. Results: The behavioral results indicate that personality trait words that are semantically congruent with attractiveness levels facilitate the perception of attractiveness, whereas incongruent trait information produces the opposite effect. Event-related potentials revealed that the influence of personality trait words on facial attractiveness was primarily related to motivated attention as indicated by the late positive component. In the voice task, personality trait words impacted vocal attractiveness processing first during semantic integration (N400 component) and then modulated motivated attention. Conclusions: These results suggest that alleged personality traits modify attractiveness processing in faces and voices in relatively late and partially modality-specific stages.

## 1. Introduction

Attractiveness refers to the degree to which a person’s sexual appeal or beauty captures the perceiver’s attention and elicits positive emotional experiences and a desire to approach [1,2]. Individuals who are physically attractive or have an appealing voice are commonly believed to possess more socially desirable, positive personality traits, reflecting the stereotype of “what is beautiful is good” [3,4]. In turn, personality traits can also influence evaluations of facial and vocal attractiveness; that is, individuals with positive personality traits are often perceived as more attractive, aligning with the stereotype of “what is good is beautiful” [5,6]. This stereotype represents the reverse mechanism of the halo effect, evolving from the notion that “what is beautiful is good”, which is supported by meta-analytic evidence [7]. People may infer others’ personality traits through external features such as facial, vocal, and body attractiveness. Similarly, alleged personality traits, such as morality (which is often considered a dimension of personality traits in social perception contexts [8]), can influence the perceived attractiveness of external features. However, the perceptual or cognitive processes through which personality trait information influences perceived attractiveness are not well understood. By examining how personality traits modulate perceptions of attractiveness across facial and vocal modalities, this work seeks to expand our understanding of the cognitive and neural mechanisms underlying these stereotypes. The present study investigated this question combining a Stroop-like paradigm with the event-related potential (ERP) technique.

### 1.1. The Perception of Facial Attractiveness and Its Relationship with Personality Traits

ERP studies have shown that facial attractiveness affects brain responses such as the P1, N170, P2, and LPC components [9,10,11,12], with highly attractive faces eliciting larger N170 compared to less attractive faces [10], indicating more pronounced structural encoding of faces and motivated attention. Additionally, interaction with attractive individuals or its anticipation triggers positive emotional responses and a desire for a closer relationship [13].

Facial attractiveness influences various social and psychological outcomes, including mate selection, kin relationships and career prospects [14] and the inference of certain personality traits. For example, there is the “what is beautiful is good” stereotype, where attractive individuals are attributed more positive behaviors, characteristics, and personality traits and are treated more favorably [5]. Furthermore, teachers’ evaluations of students across multiple dimensions, such as their academic potential and intelligence, and students’ evaluations of teachers in areas, such as organizational skills and classroom management, are influenced by attractiveness levels [15]. Preferences for attractiveness also exist in the labor market, social interactions, and even in politics [16,17,18,19].

Personality traits are amongst the most important factors influencing mate selection, cooperation, and decision-making. Li et al. and Zhang et al. have shown that positive personality traits can increase the acceptance of unfair proposals and improve the rating of facial attractiveness [20,21]. Research by Little et al. and Niimi & Goto explored how individuals’ expectation of their partner’s personality traits affected their preference for faces; they found that expected personality traits could significantly affect the attractiveness evaluation of faces [22,23]. He et al. reported that faces related to prosocial moral vignettes were considered more attractive [24] and further supported the bidirectional relationship between moral character and aesthetic judgment.

### 1.2. The Perception of Vocal Attractiveness and Its Relationship with Personality Traits

ERP studies on vocal attractiveness have found that, as compared to low-attractive voices, high-attractive voices elicited larger N1 amplitudes [25,26,27] and LPC amplitudes [25,26] in both conscious vocal attractiveness judgments and passive listening. These studies suggest that vocal attractiveness processing involves early rapid and mandatory processes, as well as late, strategy-based aesthetic decisions.

Voice also plays a role in shaping impressions related to social evaluations [28]. When visual cues are absent, such as hearing voices on the radio or telephone, listeners make judgments about the speaker based on vocal information [29]. Listeners exhibit high consistency in social evaluations and personality judgments of others even when faced with different speech content or in different language contexts [30,31]. This phenomenon occurs partly because vocal acoustic information is correlated with actual personality traits [31], and we can make judgments based on non-visual cues alone (for a review, see Groyecka et al. [32]). Indeed, vocal acoustic characteristics influence personality trait attribution, such as trustworthiness [33]. During first impression formation, vocal attractiveness was positively correlated with judgments of the speaker’s confidence, openness, and conscientiousness [34]. For example, when experts are asked to provide professional advice, they lower their average pitch and vocal tract resonance, presumably in order to obtain favorable evaluations in social environments [32].

Research on the influence of ascribed personality traits on vocal attractiveness is relatively scarce. One study examined the bidirectional relationship between vocal attractiveness and morality in the impression formation process [6]. The results revealed a tendency to rate the voices of individuals associated with positive moral impressions as more attractive, in addition to associating highly attractive voices with better moral qualities. While these findings illustrate a harmonious bidirectional influence when perceptual and semantic cues align, a key question remains: How are evaluations formed when vocal attractiveness and ascribed personality traits provide conflicting signals, such as an appealing voice paired with negative moral descriptors?

One useful framework for understanding this apparent contradiction is provided by cognitive conflict and conflict-monitoring theories [35,36]. This framework posits that conflicts in information processing—such as mismatches between perceptual attractiveness signals and semantic trait valence—trigger an evaluative monitoring system (potentially involving the anterior cingulate cortex) to detect the demand for control, leading to compensatory adjustments in attention and integration. According to this account, conflict can arise when concurrently active representations imply different evaluations—in our study, when externally derived perceptual signals (e.g., voice or face attractiveness) and internally represented semantic trait information favor different responses. When both types of cues are presented simultaneously—for example, a perceptually attractive face paired with a negative personality descriptor—these inputs create a representational mismatch that triggers conflict detection and resolution processes [35,37]. This framework predicts that external cues, given their perceptual salience, often dominate initial impressions, while internal cues exert stronger influence when perceptual information is ambiguous or uncertain. Neurocognitively, this conflict–resolution process is indexed by distinct ERP signatures: semantic mismatch arising from internal trait information is reflected in N400 modulations [38], whereas later evaluative re-processing and control-related adjustments are reflected in late positive potentials (LPCs) associated with motivated attention [39]. In this way, conflict-monitoring theory provides a mechanistic account of how external perceptual signals and internal personality information are weighted and integrated in attractiveness evaluations.

### 1.3. The Present Study

Previous research has found that facial/vocal attractiveness and personality traits mutually influence each other. However, the neural mechanisms underlying the influence of (putative) personality traits on attractiveness perception are still unclear since pertinent studies are still missing. In this study, we used electroencephalography (EEG) and a Stroop-like paradigm to investigate the neurocognitive processes involved in the influence of personality traits on perceiving facial and vocal attractiveness. The classic Stroop paradigm is a widely used task to measure cognitive conflict arising from interference between relevant and irrelevant stimulus dimensions [40]. Stroop-like paradigms are variations of this classic paradigm, adapted to probe cognitive conflict in other domains, such as semantic, emotional, or cross-modal incongruencies (e.g., spatial Stroop tasks where stimulus location conflicts with content). In the present study, we employed a Stroop-like paradigm where facial or vocal stimuli of high or low attractiveness are presented simultaneously with positive or negative personality trait words, analogous to the classic Stroop task’s simultaneous presentation of conflicting dimensions. Trait words presented concurrently with face/voice stimuli are expected to automatically activate semantic associations or valuations (e.g., “honest” with positive), which can be congruent or incongruent with the perceptual attractiveness cue, thereby producing interference analogous to Stroop interference.

Our experiment included two tasks: In the face task, we presented participants with face stimuli of high or low attractiveness along with written denotations of positive or negative personality traits, requiring them to judge facial attractiveness. The voice task was similar to the face task except that voice stimuli of high or low attractiveness was presented and vocal attractiveness was to be judged. Both tasks included congruent conditions (high facial/vocal attractiveness-positive personality traits, low facial/vocal attractiveness-negative personality traits) and incongruent conditions (high facial/vocal attractiveness-negative personality traits, low facial/vocal attractiveness-positive personality traits).

In terms of behavioral outcomes, based on previous research on stereotypes, we hypothesized that personality trait words semantically congruent with the attractiveness levels would facilitate attractiveness judgments, whereas incongruent trait information would counteract these evaluations. Furthermore, as a strong signal, the face may be relatively less affected by this effect. As for the ERP results, according to previous studies [10], we predicted that, compared to low-attractive faces, high-attractive faces would elicit larger N170 and LPC amplitudes, but low-attractive faces would elicit larger P1 amplitudes compared to high-attractive faces [9]. Similarly, compared to low-attractive voices, high-attractive voices should elicit larger N1, P2, and LPC amplitudes [25,26,27]. The N400 typically begins 200–300 ms after the presentation of visual or auditory stimuli and peaks around 400 ms [41] and is thought to be triggered, among other things, by semantic conflict [42], conflict control [43] and cognitive resource allocation [44]. Based on the conflict-monitoring account and prior ERP findings, we predicted that incongruent pairings would induce greater semantic/representational conflict [42]—manifesting as enhanced N400 amplitudes—followed by increased LPC amplitudes reflecting control-related re-evaluation. Moreover, because external perceptual cues (especially faces) may be highly salient and dominate initial processing, semantic conflict may be resolved later for faces (producing stronger LPC effects), whereas vocal attractiveness—being comparatively more susceptible to semantic/contextual information—may more readily elicit early semantic conflict indexed by a larger N400 [45].

Previous research has found that gender affects attractiveness preferences [46,47]. Specifically, men are more inclined to be attracted to attractive female faces or voices, while women prefer attractive male faces or voices and, furthermore, women who prefer male faces tend to also prefer male voices [48]. Therefore, in order to reduce the complexity of the experimental design, we decided to recruit only female participants and to use only male faces and male voice stimuli.

## 2. Methods

### 2.1. Participants

Based on previous research [49], the sample size was estimated by using G*power 3.1 software [50]. We specified an F-test for an ANOVA: “Repeated measures, within factors” (type of power analysis: a priori), set the effect size to f = 0.25 (medium), α = 0.05, power (1 − β) = 0.80, number of groups = 1 (within-subjects design), and number of measurements = 4 (four within-subject conditions). The correlation between repeated measurements and spherical vacation devices was set by default (correlation among repeated measures = 0.50 and nonsphericity correction ε = 1). Under these settings, G*Power indicated that a sample of approximately 23 participants (N ≈ 23) was required. A total of 30 female college students from Liaoning Normal University were recruited. All participants reported being heterosexual, with no history of mental illness, and having normal or corrected-to-normal hearing and vision. Three participants were excluded from the behavioral data analysis due to an error rate exceeding 50%. Due to equipment failure, EEG data from two additional participants were not recorded, and EEG data from two participants were excluded due to low quality (low number of valid trials after preprocessing and excessive artifacts). Consequently, the final sample for data analysis included 23 female participants with an average age of 22.12 ± 1.92 years. The experiment was approved by the Research Ethics Committee of Liaoning Normal University. All participants read and signed an informed consent form before the experiment and received compensation thereafter.

### 2.2. Stimuli

The stimuli included voice recordings, face images, and personality trait words. All voice recordings were selected from our own database and were of the emotionally neutral Chinese words “历史 (history)”, “事物 (things)”, and “心脏 (heart)”. The recordings had been made in a quiet environment using a high-quality microphone (Bluebird SL, Blue Microphones, Westlake Village, CA, USA) and Adobe Audition CS6 3.0 software, with a sampling rate of 44,100 Hz at a bit rate of 32 kbps, and stored in wav format. Praat software (Version 4.5.16) was used for editing, retaining only the voiced segments, standardizing loudness at 70 dB, and removing any recordings with noticeable background noise, mispronunciations, or unnatural speech, retaining voiced segments without equalizing F0 or duration to preserve attractiveness cues. For the experiment, 150 voice recordings from 50 male speakers were selected.

Photographs of faces were taken from previous research [51] and partially newly taken, resulting in 80 images of adult male faces. All portraits were taken from frontal view, with eyes looking straight ahead and displaying a neutral expression. Brightness and contrast were standardized using the SHINE toolbox in MATLAB version R2023a [52], which applies histogram equalization to match luminance distributions across images. This process minimized low-level perceptual confounds (e.g., variations in lighting or contrast) while preserving the natural structural features of the faces. The images were first cropped to 260 × 300 pixels and converted to grayscale with a black background using Adobe Photoshop CS6, ensuring uniformity in external features (e.g., no hair, ears, or clothing visible).

Since the formal experiment involved only female participants evaluating male stimuli, we selected high- and low- attractive stimuli based on the ratings by female participants. A total of 23 females (aged 19–26 years, *M* = 21.75, *SD* = 2.12) who did not participate in the formal experiment rated the attractiveness of all faces and voices. The stimuli were presented using E-prime 3.0 software and rated on 7-point scales (1 = very attractive, 7 = very unattractive) based on subjective impressions. The final stimuli for the ERP experiment included 30 high-attractive (*M* = 5.46, *SD* = 0.28) and 30 low-attractive male voices (*M* = 3.29, *SD* = 0.42), as well as 30 high-attractive (*M* = 4.61, *SD* = 0.39) and 30 low-attractive male faces (*M* = 2.15, *SD* = 0.22). Statistical testing confirmed the differences between high- and low-attractive stimuli for both voices (*t* = 23.38, *p* < 0.001) and faces (*t* = 29.77, *p* < 0.001).

Personality trait words (adjectives, e.g., “honest”, “evil”) were partially selected from standardized personality trait databases [53] and partially from online sources, resulting in a total of 235 two-character Chinese words. Personality trait words were rated by a mixed-gender sample, as these descriptors are not gender-specific and our aim was to obtain normative semantic valence ratings. Forty participants (21 females, 19 males, aged 19–26 years, *M* = 21.89, *SD* = 2.12) were recruited to rate each word on valence, familiarity, and gender typicality. In the valence rating task, 1 indicated a very negative word, 4 indicated neutral, and 7 indicated very positive. In the familiarity rating task, 1 indicated very unfamiliar, 4 indicated moderate familiarity, and 7 indicated very familiar. In the gender typicality rating task, 1 indicated that the word described only females, 4 indicated that the word could describe both males and females, and 7 indicated that the word described only males. The mean and standard deviation were calculated for the valence, familiarity, and gender typicality dimensions. Finally, 90 negative words (valence: *M* = 2.55, *SD* = 0.34, familiarity: *M* = 5.11, *SD* = 0.22, gender typicality: *M* = 3.93, *SD* = 0.29) and 90 positive words (valence: *M* = 5.72, *SD* = 0.25, familiarity: *M* = 5.70, *SD* = 0.22, gender typicality: *M* = 4.00, *SD* = 0.32) were selected as the final experimental materials. Statistical testing confirmed the differences between the negative and positive words for valence (*t* = −72.13, *p* < 0.001), familiarity (*t* = −18.05, *p* < 0.001), and gender typicality (*t* = −1.48, *p* = 0.14).

### 2.3. Procedure

The experiment was conducted in a room with soft lighting, quiet and comfortable surroundings, and electromagnetic shielding. Participants sat in front of a computer monitor at 75 cm. The monitor size was 17 inches with a resolution of 1920 × 1080 pixels; stimuli subtended visual angles of approximately 5°. Auditory stimuli were presented through headphones at a subjectively comfortable volume. Each participant completed two Stroop-like tasks: one for facial attractiveness and one for vocal attractiveness judgements, and they were asked to classify the face or voice as attractive or unattractive according to their own criteria (they were not informed about the preselection of stimuli) while ignoring the personality words. In the task, faces/voices and personality trait words were presented simultaneously to induce automatic semantic activation and thereby probe semantic interference in attractiveness judgments. Participants were to quickly and spontaneously (without much deliberation) classify each face/voice according to attractiveness.

**Figure 1:**
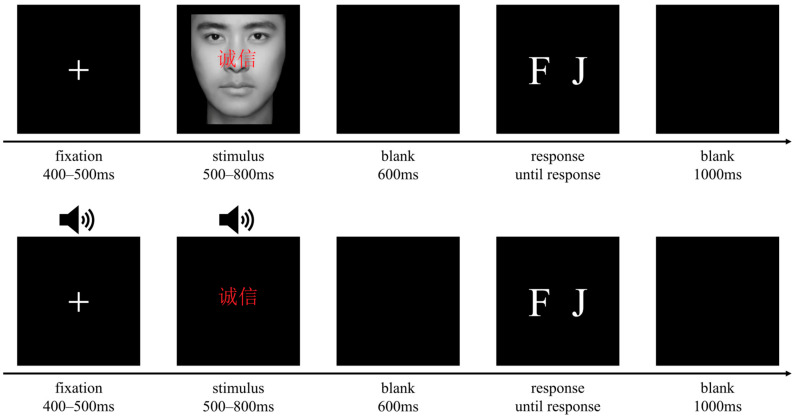
Trial scheme of the face and voice task (top vs. bottom).

As shown in Figure 1, each trial began with a fixation point (accompanied by a “beep” sound in the voice task) that lasted 400–500 ms. This was followed by the simultaneous presentation of a face (or voice) and a personality trait word for 500–800 ms, with the personality words positioned above the nose of the face (at the same location as in the voice task). Participants were instructed to withhold any movement during the stimulus and blank screen, and only after the 600 ms blank screen were they required to classify the attractiveness of each face/voice by pressing the “F” or “J” key on the keyboard, with key assignments counterbalanced between participants. A final 1000 ms blank screen was presented before the next trial began.

All face and voice stimuli were presented across four blocks, with two blocks each for faces and voices; the order of face and voice tasks was counterbalanced across participants. In both tasks, stimuli were pseudo-randomly assigned to two blocks, each containing 90 trials. The paired stimuli yielded four conditions with 45 trials each (high-attractive voices/positive traits, high-attractive voices/negative traits, low-attractive voices/positive traits, low-attractive voices/negative traits). A practice phase with 16 additional trials was included to ensure that participants understood the experimental procedure. The entire experiment took approximately one hour, including preparation, practice, and the main experiment.

### 2.4. Data Collection and Analysis

The EEG signals were recorded using a 64-channel Ag/AgCl electrode cap (Brain Products, Gilching, Germany) based on the extended 10–20 international system. Continuous EEG signals were recorded by using FCz as common online reference, a 100 Hz low-pass filter and a sampling rate of 500 Hz. The impedance of all scalp electrodes was maintained below 5 kΩ. During offline analysis, the data were re-referenced to the averaged mastoid channels using EEGLAB v2023.1 (MathWorks, Natick, MA, USA). Data were preprocessed with a bandpass filter of 0.01 Hz (24 dB/octave) to 30 Hz (24 dB/octave). Obvious artifacts such as EMG and head movements were manually removed. Further artifact correction was performed using independent component analysis (ICA) [54], and trials with amplitudes exceeding ±100 μV were excluded. Components were visually inspected and rejected if they clearly reflected ocular artifacts (e.g., blinks or eye movements, characterized by frontal topography and slow waveforms), muscular artifacts (e.g., high-frequency noise with peripheral topography), or other non-neural sources (e.g., line noise or electrode pops). The EEG data were segmented from 200 ms before to 800 ms after the onset of the face or voice stimuli, with a baseline correction based on the 200 ms pre-stimulus period. Only trials with participant responses consistent with the pre-experimental ratings were retained for ERP averaging. This was the case in 74.1% of trials in total.

In the ERPs of both face and voice tasks, the N400 component was measured in the 300–500 ms time window at a frontal-central ROI (FCz, FC1, FC2, FC3, FC4, Cz, C1, C2, C3, C4) [42,55]. The LPC typically shows a parietal scalp distribution (for attractive relative to non-attractive faces) [12,56] and voices [45] but with some possible variance. In the present results, we found that the differences for attractive and non-attractive stimuli in the late ERPs differed in topographies and time windows in face and voice tasks. Therefore, in both the face and voice tasks, the LPC component was analyzed in two consecutive time windows: 500–650 ms and 650–800 ms. For the face task, mean amplitudes were extracted from a centro-parietal ROI (CP1, CP2, CPz, P1, P2, Pz); whereas for the voice task, a fronto-central ROI (FCz, FC1, FC2, FC3, FC4, Cz, C1, C2, C3, C4) was used. It should be noted that the late window of LPC is within the interval of the blank screen, but it ends still more than 300 ms before the fastest response.

Additionally, in the face task ERPs we measured the P1 in the 90–140 ms time window at an occipito-temporal ROI (O1, Oz, O2) [9] and the N170 component during the 160–220 ms time window at an occipito-temporal ROI (O2, O1, Oz, POz, PO8, PO7, PO4, PO3) [57]. In the voice task ERPs, the N1 component was measured in the 90–140 ms time window at the fronto-central region of interest (ROI: F1, Fz, F2, FC1, FCz, FC2, C1, Cz, C2) [10,27]; the P2 component was measured during the 160 to 230 ms time window at a fronto-central ROI (F2, F1, Fz, FC2, FC1, FCz, Cz, C1, C2) [58].

ERP amplitudes were statistically analyzed using repeated measures ANOVAs with factors Attractiveness (high attractiveness, low attractiveness) and Congruency (congruent, incongruent). Our analytic strategy followed a theory-driven ROI approach: each ERP component/time window was pre-specified based on prior literature, and Bonferroni correction was applied in SPSS v27.0.0 for post hoc pairwise comparisons following significant ANOVA effects. This was carried out for each component to control Type I error in targeted comparisons.

## 3. Results

According to Liu et al. [45], for both the face and voice task, performance was evaluated in terms of percent agreement with the rating-based attractiveness categories, distinguishing between attractive and non-attractive stimuli and whether they were congruent or incongruent with the personality trait words. The primary behavioral measure was “percent agreement” with the pre-experimental attractiveness ratings because participants judged attractiveness subjectively (based on their personal taste, without awareness of the pre-ratings. Percent agreement was calculated as the proportion of trials where participants’ classifications matched the pre-rated category (high = attractive, low = unattractive), computed per condition. To simplify the results and exclude irrelevant factors, only trials consistent with the attractiveness ratings of the stimuli were included in the subsequent ERP statistical analysis. Additionally, to ensure that personality traits and attractiveness processing were not influenced by keypresses, participants were required to make classifications 600 ms after stimulus presentation and reaction times were not analyzed. The order of the face and voice tasks was counterbalanced across participants (approximately half of participants completed the face task first and half completed the voice task first). Task order was controlled at the design stage but was not included as an explicit factor in the reported ANOVAs. In the following, we will report the results of the two tasks separately, mainly because ERPs to faces and voices differ in their basic morphology—due to the modality difference—and are therefore hard to compare.

### 3.1. Performance Results

#### 3.1.1. Face Task

A repeated measures ANOVA was conducted to analyze the effects of Attractiveness and Congruency. The main effect of Attractiveness was significant, *F*(1,22) = 42.96, *p* < 0.001, *η_p_*^2^ = 0.661, with higher percent agreement of participants’ classifications with the pre-ratings for low attractive faces (*M* = 0.954, *SD* = 0.018) than for high attractive faces, (*M* = 0.802, *SD* = 0.024). The main effect of Congruency was also significant, *F*(1,22) = 9.25, *p =* 0.006, *η_p_*^2^ = 0.296, with higher percent agreement with the pre-ratings under the congruent condition (*M* = 0.907, *SD* = 0.019) being higher than under the incongruent condition (*M* = 0.85, *SD* = 0.021).

**Figure 2:**
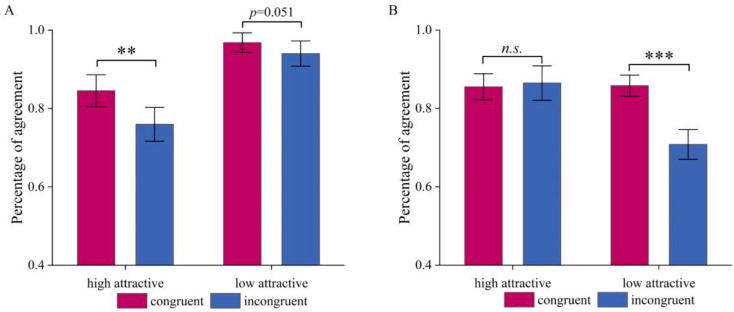
Percent agreement of attractiveness judgments with the pre-experimental attractiveness rating categories. (**A**) Attractiveness judgment in the face task. (**B**) Attractiveness judgment in the voice task. Only interaction effects are marked. ** *p* < 0.01, *** *p* < 0.001, *^n.s.^ p* > 0.01.

In addition, the interaction between Attractiveness and Congruency was significant, *F*(1,22) = 7.87, *p =* 0.01, *η_p_*^2^ = 0.264. Further simple effects analyses revealed that for low-attractive faces, the main effect of Congruency exhibited a strong trend toward significance, *F*(1,22) = 4.27, *p =* 0.051, *η_p_*^2^ = 0.163, with higher percent agreement with the pre-ratings in the congruent condition (*M* = 0.968, *SD* = 0.016) than in the incongruent condition (*M* = 0.94, *SD* = 0.021). For high-attractive faces, this effect was even more pronounced and significant, *F*(1,22) = 10.09, *p =* 0.004, *η_p_*^2^ = 0.315, with higher percent agreement with the pre-ratings in the congruent condition (*M* = 0.845, *SD* = 0.027) than in the incongruent condition (*M* = 0.759, *SD* = 0.029), see Figure 2A.

#### 3.1.2. Voice Task

In the voice task the main effect of Attractiveness was significant, *F*(1,22) = 5.14, *p =* 0.034, *η_p_*^2^ = 0.189, with higher percent agreement with the pre-ratings for high attractive voices (*M* = 0.86, *SD* = 0.023) than for low attractive voices, (*M* = 0.783, *SD* = 0.02). The main effect of Congruency was also significant, *F*(1,22) = 14.02, *p =* 0.001, *η_p_*^2^ = 0.389, with better percent agreement with the pre-ratings in the congruent condition (*M* = 0.857, *SD* = 0.012) than in the incongruent condition (*M* = 0.786, *SD* = 0.019).

More importantly, also the interaction between Attractiveness and Congruency was significant, *F*(1,22) = 45.11, *p* < 0.001, *η_p_*^2^ = 0.672. Simple effects analyses revealed that for the low-attractive voices, the main effect of Congruency was significant, *F*(1,22) = 67.11, *p* < 0.001, *η_p_*^2^ = 0.753, with percent agreement with the pre-ratings in the congruent condition (*M* = 0.858, *SD* = 0.018) higher than in the incongruent condition (*M* = 0.708, *SD* = 0.025). In contrast, for high-attractive voices, we did not find an effect of Congruency (*p* = 0.708; see Figure 2B).

### 3.2. ERP Results

#### 3.2.1. Facial Attractiveness Classification Task

**Figure 3:**
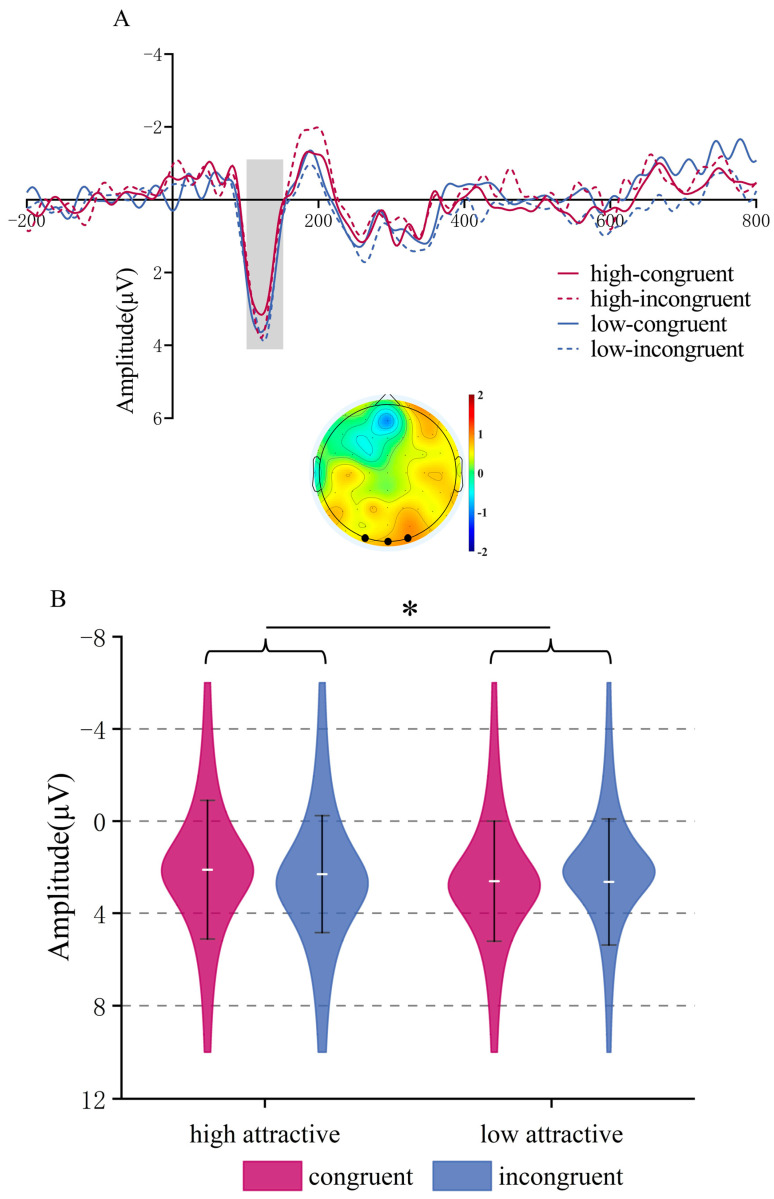
P1 component in the Facial Attractiveness Classification task. (**A**) The ERP waveforms in the region of interest (ROI; see bold points in the topography) for the P1 component, along with topographical maps showing the low-attractive condition minus the high-attractive condition within the 90–140 ms time window. (**B**) The ERP amplitudes of the P1 component in each condition (White lines are averages, same as below). * *p* < 0.05.

**P1.** The statistical results for the P1-component in the face task revealed a significant main effect of Attractiveness *F*(1,22) = 4.89, *p =* 0.038, *η_p_*^2^ = 0.182. Further analysis showed that low-attractive faces induced greater P1 amplitude than high-attractive faces, (*M* = 2.625, *SD* = 0.529 μV) vs. (*M* = 2.208, *SD* = 0.55 μV). In addition, the main effect of Congruency (*p* = 0.578) and the interaction between Attractiveness and Congruency (*p* = 0.789) were not significant, see Figure 3.

**Figure 4:**
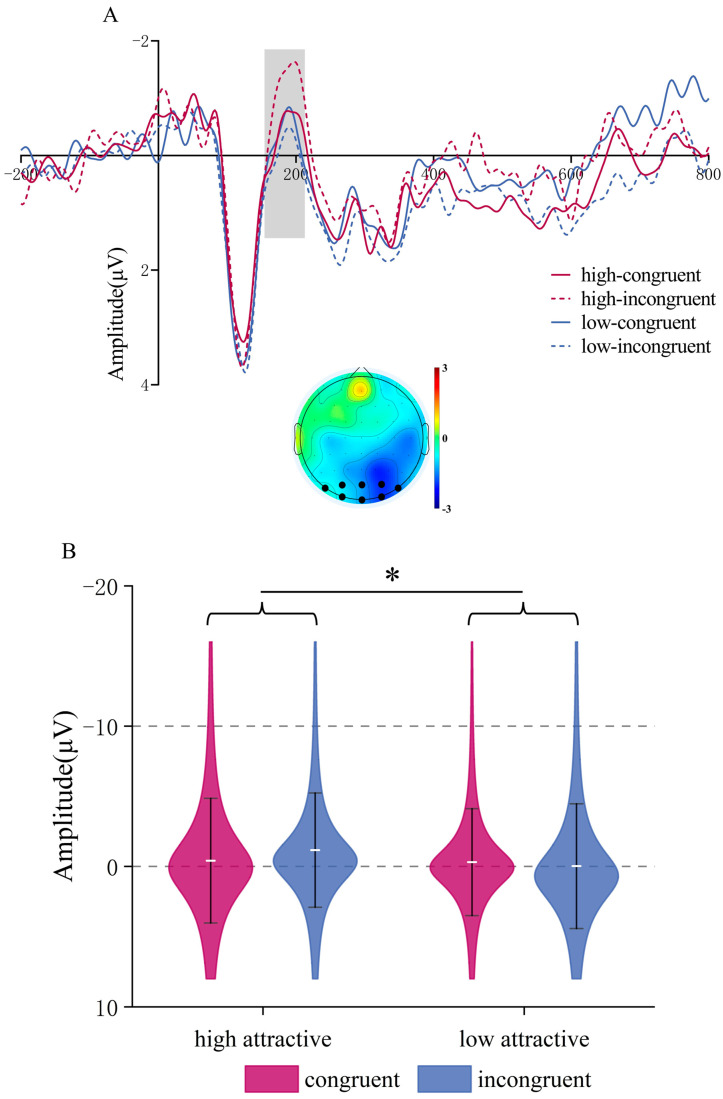
N170 component in the Facial Attractiveness Judgment task. (**A**) The ERP waveforms in the region of interest (ROI) for the N170 component, along with topographical maps showing the high attractive condition minus the low attractive condition within the 160–220 ms time window. (**B**) ERP amplitudes of the N170 component for each condition. * *p* < 0.05.

**N170.** ANOVA revealed a significant main effect of Attractiveness, *F*(1,22) = 7.18, *p =* 0.014, *η_p_*^2^ = 0.246, with high-attractive faces eliciting a more negative (larger) N170 component compared to low-attractive faces, (*M* = −0.786, *SD* = 0.862 μV) vs. (*M* = −0.164, *SD* = 0.846 μV). The main effect of Congruency (*p* = 0.338) and the interaction between Attractiveness and Congruency was not significant (*p* = 0.108), see Figure 4.

**N400**. The statistical results for this component showed no significant main effects (Attractiveness, *p* = 0.264; Congruency, *p* = 0.975) or interaction, *p* = 0.915.

**LPC (500–650 ms time window)**. The statistical results for this time window showed no significant main effects (Attractiveness, *p* = 0.271; Congruency, *p* = 0.695) or interaction, *p* = 0.95.

**Figure 5:**
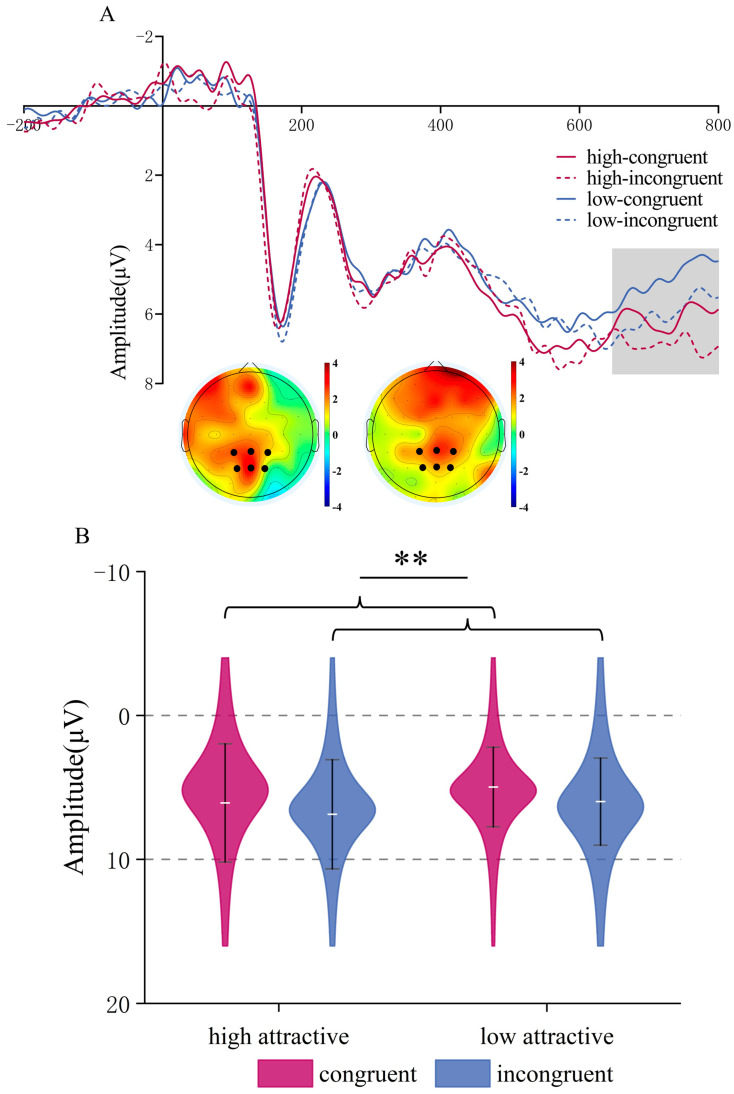
LPC Component in Facial Attractiveness Judgment. (**A**) The ERP waveforms in the region of interest (ROI) for the LPC component, along with topographical maps showing the high attractive condition minus the low attractive condition (**left**) and the incongruent condition minus the congruent condition (**right**) within the 650–800 ms time window. (**B**) ERP amplitudes of the LPC component for each condition combination. ** *p* < 0.01.

**LPC (650–800 ms time window)**. There was a trend for a main effect of Attractiveness, *F*(1,22) = 3.58, *p* = 0.072, *η_p_*^2^ = 0.14; high-attractive faces elicited larger amplitudes than low-attractive faces (*M* = 6.471, *SD* = 0.744 μV) vs. (*M* = 5.48, *SD* = 0.558 μV)). The main effect of Congruency was significant, *F*(1,22) = 9.97, *p* = 0.005, *η_p_*^2^ = 0.312, incongruent face/word stimuli induced greater LPC amplitude than congruent stimuli, (*M* = 6.426, *SD* = 0.598 μV) vs. (*M* = 5.524, *SD* = 0.641 μV). The interaction was not significant (*p* = 0.835), see Figure 5.

#### 3.2.2. Vocal Attractiveness Classification Task

**Figure 6:**
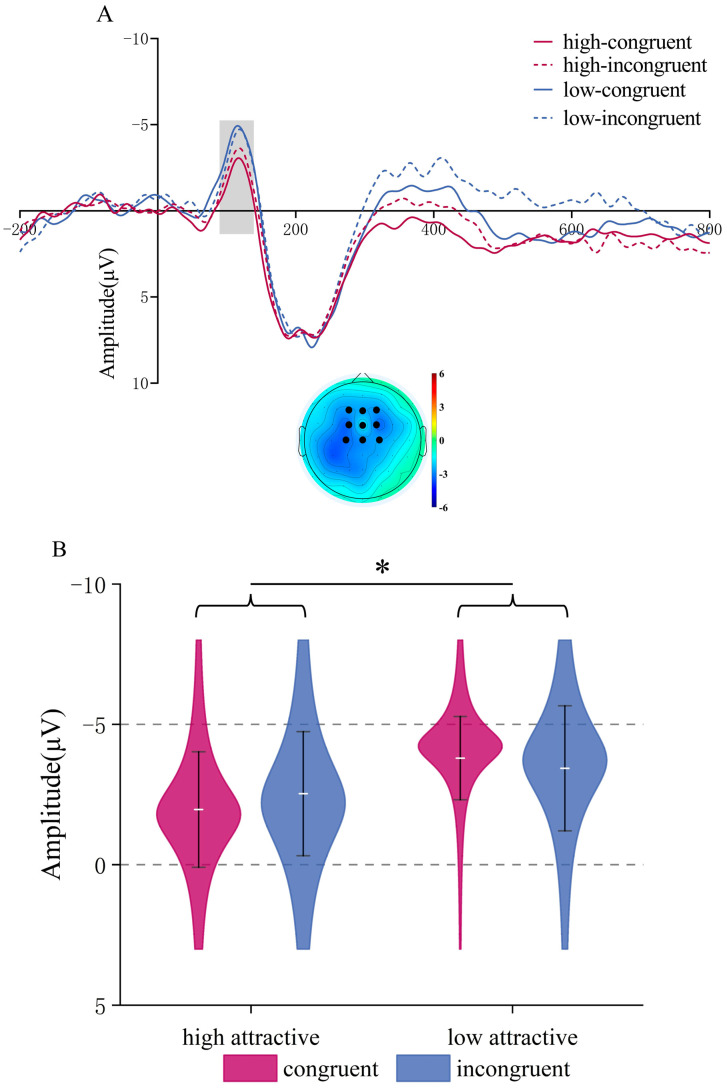
N1 component in the Vocal Attractiveness Judgment task. (**A**) Grand mean ERP waveforms of the N1 (ROI) during classification of vocal attractiveness, and the difference topography of the low attractive minus the high attractive conditions in the 90–140 ms time window. (**B**) ERP amplitudes of the N1 component for four condition combinations. * *p* < 0.05.

**N1**. The ANOVA revealed a significant main effect of Attractiveness, *F*(1,22) = 16.17, *p* < 0.001, *η_p_*^2^ = 0.424, with low-attractive voices eliciting larger N1 amplitudes (*M* = −3.615, *SD* = 0.344 μV) than high-attractive voices (*M* = −2.248, *SD* = 0.391 μV). However, the main effect of Congruency (*p* = 0.652) and the interaction between Attractiveness and Congruency (*p* = 0.188) were not significant. See Figure 6.

**P2**. The statistical results showed no significant main effects (Attractiveness, *p* = 0.268; Congruency, *p* = 0.699) or interaction, *p* = 0.905.

**Figure 7:**
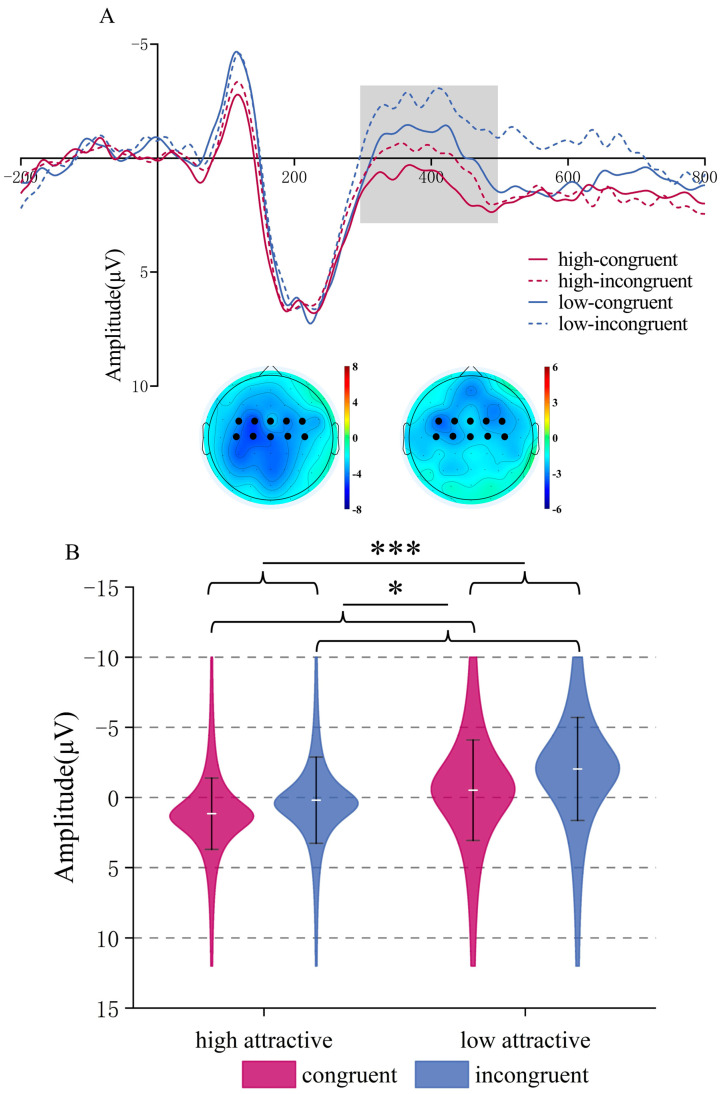
N400 component in the Vocal Attractiveness Judgment task. (**A**) Grand mean ERP waveforms for the N400 ROI during vocal attractiveness classification, and the difference topography of the low attractive minus high attractive voice condition (**left**) and the incongruent minus congruent condition (**right**) in the 300–500 ms time window. (**B**) ERP amplitude of the N400 component under different conditions. * *p* < 0.05, *** p < 0.001.

**N400.** ANOVA indicated a significant main effect of Attractiveness, *F*(1,22) = 38.64, *p* < 0.001, *η_p_*^2^ = 0.637; low-attractive voices induced more negative N400 amplitudes (*M* = −1.277, *SD* = 0.687 μV) compared to high-attractive voices (*M* = 0.675, *SD* = 0.541 μV). The main effect of Congruency was also significant, *F*(1,22) = 6.77, *p* = 0.016, *η_p_*^2^ = 0.235, with more negative N400 amplitudes in the incongruent condition (*M* = −0.92, *SD* = 0.688 μV) than in the congruent condition (*M* = 0.317, *SD* = 0.596 μV). There was no interaction (*p* = 0.343), see Figure 7.

**LPC (500–650 ms time window)**. The ANOVA showed a significant main effect of Attractiveness, *F*(1,22) = 14.77, *p* < 0.001, *η_p_*^2^ = 0.402; high-attractive voices induce larger LPC amplitudes compared to low-attractive voices, (*M* = 1.651, *SD* = 0.486 μV) vs. (*M* = 0.253, *SD* = 0.652 μV). The main effect of Congruency was also significant, *F*(1,22) = 5.82, *p* = 0.025, *η_p_*^2^ = 0.209, congruent trait/voice combinations induced more positive LPC amplitudes compared to incongruent combinations, (*M* = 1.505, *SD* = 0.623 μV) vs. (*M* = 0.398 ± 0.558 μV).

**Figure 8:**
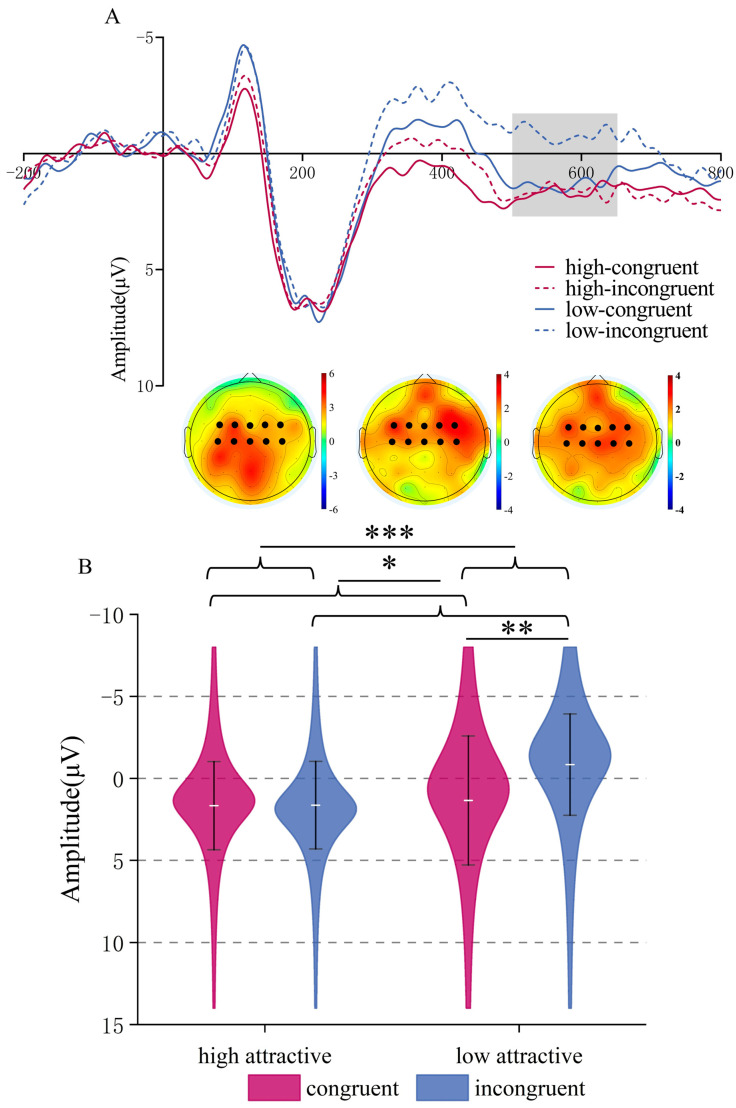
LPC component in the Vocal Attractiveness Judgment task. (**A**) Grand mean ERP waveforms for the LPC ROI during vocal attractiveness classification, and the difference topography of the high attractive voice condition minus the low attractive voice condition (**left**), the congruent condition minus the incongruent condition (**middle**), and the low attractive-congruent condition minus the low attractive-incongruent condition (**right**) in the 500–650 ms time window. (**B**) ERP amplitude of the LPC component under different conditions. * *p* < 0.05, ** *p* < 0.01, *** *p* < 0.001.

Importantly, there was a significant interaction between Attractiveness and Congruency, *F*(1,22) = 6.43, *p* = 0.019, *η_p_*^2^ = 0.226. Simple effects analysis indicated that for low-attractive voices, congruent trait words induced larger LPC amplitudes than incongruent words, *F*(1,22) = 9.96, *p* = 0.005, *η*_p_^2^ = 0.312, (*M* = 1.345, *SD* = 0.82 μV) vs. (*M* = −0.839, *SD* = 0.645 μV); in contrast, for high-attractive voices, there was no significant effect of congruency (*p* = 0.956), see Figure 8.

**Figure 9:**
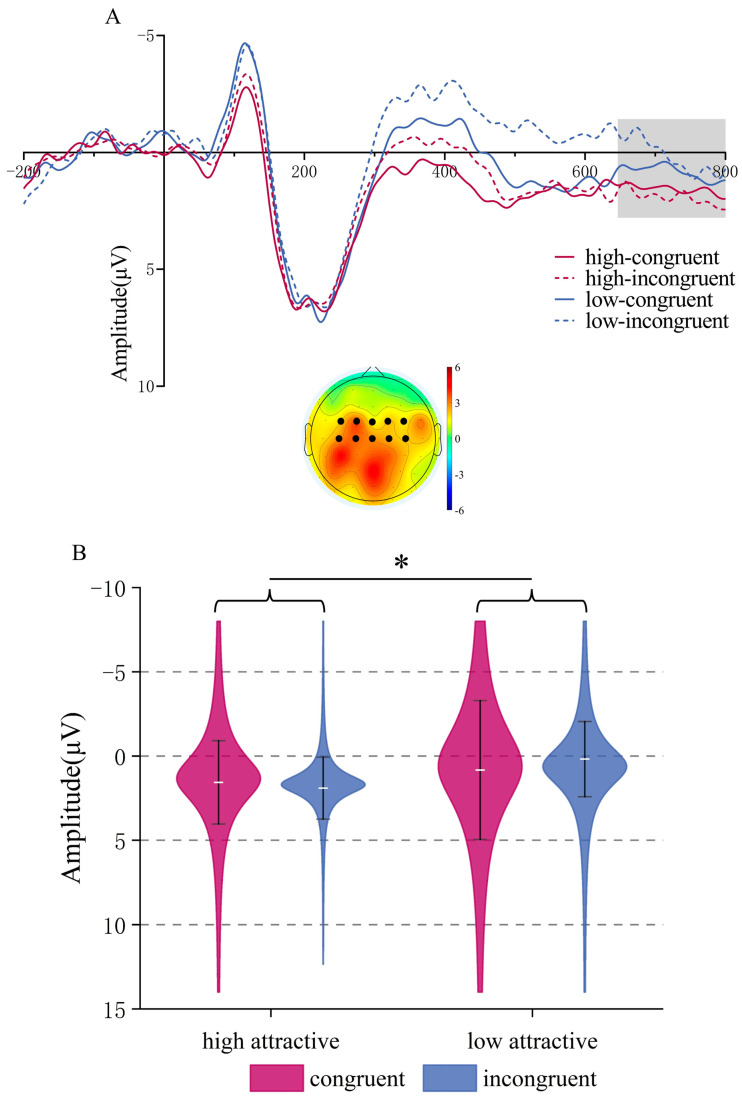
(**A**) Grand mean ERP waveforms for the LPC ROI during vocal attractiveness classification, and the difference topography of the high attractive minus the low attractive voice condition in the 650–800 ms time window. (**B**) ERP amplitude of the LPC component under different conditions. * *p* < 0.05.

**LPC (650–800 ms time window)**. For this time window ANOVA showed a significant main effect of Attractiveness, *F*(1,22) = 6.77, *p* = 0.016, *η_p_*^2^ = 0.235, with a more positive LPC for high attractive than low attractive voices, (*M* = 1.735, *SD* = 0.348 μV) vs. (*M* = 0.507, *SD* = 0.593 μV). The main effect of Congruency (*p* = 0.751) and the interaction (*p* = 0.267) were not significant. See Figure 9.

## 4. Discussion

Using ERP technology in a Stroop-like paradigm we examined the effects of positive or negative associated personality traits on the classification of facial and vocal attractiveness. The behavioral and ERP results show that personality traits modulate perception of attractive levels and show differences in face and voice tasks. At the behavioral level, we found that the influence of personality trait words on perceived attractiveness differed between facial and vocal stimuli. Analysis of the EEG data further revealed the temporal dynamics underlying this effect. In fact, the influence of personality traits on attractive judgments is not expressed in isolation but emerges through their interaction with perceptual cues. It is this interplay—where trait information can either reinforce or conflict with facial/voice attractiveness—that reveals the dynamic contribution of personality to impression formation. In the following, we will discuss these findings in greater detail.

### 4.1. Performance Evidence

Significant main effects of congruency revealed that agreement of participants’ attractiveness ratings with the corresponding stimulus categories was higher when personality traits were congruent rather than incongruent with the stimulus category. This result consistent with the “what is good is beautiful” stereotype, where positive traits enhance perceived attractiveness and negative traits diminish [3].

In the face task, percentage of agreement for low-attractive faces was significantly higher than for high-attractive faces; in the voice task, it was higher for high- than low-attractive voices. First, people’s opinions on unattractive faces may tend to be more consistent [59]. Moreover, this cross-modal asymmetry cannot be attributed to a single mechanism. Although low-attractive voices and high-attractive faces were less concordant than their intra-modal counterparts, this likely reflects their closer proximity to the neutral mid-point on pre-experimental ratings, rather than inherent valence effects. Such extremity or ambiguity differences may modulate the impact of top-down personality information on judgments. Prior work suggests moderate or ambiguous attractiveness is more susceptible to contextual or trait-based modulation [5,60]. However, in the present study, the asymmetry results may be influenced by both the valence direction (high vs. low) and stimulus extremes (extreme vs. moderate), which cannot be clearly distinguished by the current design. Thus, we refrain from strong causal claims and recommend future studies equating modality extremes or using pre-ratings as continuous covariates.

The interaction effect revealed that the congruency effect was stronger in the high-attractive face condition and the low-attractive voice condition. A stronger consistency effect for highly attractive faces suggests that when the expected positive traits do not match the personality trait words presented, it more significantly disrupts the processing of attractive faces. This may be due to stronger stereotypes or expectations associated with attractive individuals [3]. Negative trait words led to reduced classification agreement for these faces, indicating cognitive conflict. In contrast, in the voice task, the distinct acoustic features of attractive voices may have aided participants in their classification. For low-attractive voices, the percentage of agreement decreased under inconsistent conditions, suggesting that consistent trait words provided more critical information for judging attractiveness. Meanwhile, high-attractive voices remained relatively stable across conditions. These findings may indicate that low-attractive voices may be relatively ambiguous and therefore rely more heavily on semantic context to guide judgments.

Overall, these findings show that semantically congruent trait information facilitates attractiveness judgments, whereas incongruent traits distort them. This effect is modulated by the level of attractiveness, highlighting the interplay between perceptual input and contextual meaning in social evaluation.

### 4.2. Effect of Personality Traits on the Perception of Facial Attractiveness

In our study, we found that low-attractive faces elicited larger P1 amplitudes than high attractive faces. This result is consistent with results of Halit et al. [9], which showed that facial morphology is processed at a very early stage, and that unattractive and atypical faces elicit larger P1 amplitudes than attractive and typical faces. The amplitude of P1 is generally thought to reflect the initial perception and processing of visual stimuli, especially in the allocation of attention and early analysis of external visual features. This early effect may reflect low-level perceptual differences, such as atypical facial features or asymmetry in low-attractive faces, which draw attention due to their deviation from prototypical face templates [2]. Then, we found that high-attractive faces induced larger N170 amplitudes compared to low-attractive faces. The N170 component has been found to be independent of facial expression in some studies, sensitive to face processing, and attractive faces may cause greater amplitudes associated with structural encoding and recognition memory [10,11,45]. The current result is consistent with this previous research. In the early P1 and N170 components, we only found a main effect of attractiveness. This is consistent with the idea that the perceptual processing of attractiveness (visual/auditory) occurs before the integration of personality trait words in a semantic manner.

In the face task, no effect was observed on the N400 component, which is noteworthy. Given that the N400 is typically linked to semantic processing, this may suggest that there was no effect of personality trait words on first-pass semantic integration or memory retrieval. In addition, the visual salience of faces dominates early processing, delaying the integration of personality traits until later stages, potentially due to the holistic nature of face perception [2].

The LPC segment was divided into two phases. In the early LPC phase, no main effects or interaction were observed. However, in the later phase, the LPC revealed a trend of larger amplitudes for highly attractive faces as well as a significant effect of consistency, with inconsistent conditions eliciting greater amplitudes. First, we should clarify that in the late LPC window, although part of it falls into the presentation interval of the blank screen, the resulting effect is materially different from the preparation potential [61,62]. Specifically, this effect exhibits a central-parietal lobe topology rather than the unilateral central distribution expected in movement preparation. Moreover, its timing is modality-specific (the LPC in the voice task occurs at 500–650 ms but at 650–800 ms in the face task), which is inconsistent with the prevailing input modality-unspecific interpretation of movement potentials. Therefore, we believe that the LPC effect is not an action preparation potential; nevertheless, this should be avoided as much as possible in subsequent studies. The long latency of the effect may be because the influence of personality traits on faces is mainly produced in later processing stages compared with voice. The marginal effect of attractiveness on the LPC is consistent with previous studies [20,26]. However, the significant LPC congruency effect—greater amplitude in incongruent than congruent conditions—deviates from previous studies. High-attractive stimuli provide profound intrinsic rewards [63,64], leading to larger LPC amplitudes in both face and voice tasks. However, it can be seen from our results that personality traits exert less influence on face than voice attractiveness perception. One possible explanation is that facial information dominates early processing, delaying the integration with personality traits. As processing unfolds, personality information gains weight, particularly when it conflicts with stereotypical expectations. This mismatch may require greater cognitive resources at later stages (reflected in LPC), consistent with top-down conflict resolution. Compared to voice attractiveness, facial attractiveness processing appears less sensitive to personality traits during early stages, possibly due to holistic face processing, which prioritizes visual features before integrating semantic cues. This delayed integration still aligns with the “what is good is beautiful” stereotype [3], but emerges later in the temporal sequence of processing.

### 4.3. Effect of Personality Traits on the Perception of Vocal Attractiveness

In the vocal attractiveness judgment task, the N1 component served as an early indicator of auditory attention toward target stimuli [65]. N1 amplitude was larger to low- than to high attractive voices, suggesting that in the initial stages of cognitive processing, individuals paid greater attention to less attractive voices. Alternatively, it could also reflect the greater difficulty to categorize low-attractive voices, which were closer to the neutral point than high-attractive voices.

Different from the face task, personality traits influence voice attractiveness, which is manifested in the N400 component. The N400 is generally considered to reflect the processing of mismatched semantic information or retrieval of semantic information [38,42,66], focusing on the consistency of stimulus conditions rather than the type of stimulus [55]. Other meaningful stimuli, such as faces [67], can also elicit this component. Most studies found that incongruent conditions elicited larger N400 amplitudes compared to congruent conditions [68]. Our results are consistent with those studies, which may be due to participants having specific impression for attractive or unattractive voices, with the simultaneous personality trait information being inconsistent with their impression, causing greater N400 amplitudes. In addition to integration processing, the N400 is related to conflict control [43], and cognitive resource allocation [44]; the increased cognitive resources needed to resolve conflicting information may lead to larger N400 amplitudes. In our study, participants’ task was to assess vocal attractiveness, which may involve suppressing conflicting stimuli unrelated to personality trait information [69,70]. Thus, the larger N400 amplitude observed under inconsistent conditions may indicate that personality trait information interfered with the attractiveness evaluation process.

For the LPC of the 500–650 ms time window, we found that it was modulated by attractiveness; high-attractive voices induced larger LPC amplitudes compared to low-attractive voices. Previous research has shown that attractive voice or face stimuli elicit larger LPC amplitudes compared to unattractive stimuli [20,26,45,49], and high- and low-attractive stimuli induced larger LPC amplitudes compared to moderately attractive stimuli [71]. High-attractive voices have greater intrinsic reward value [63,64], thus capturing more of the participant’ attention resources, resulting in larger LPC amplitudes for high-attractive voices. We further observed the main effect of congruency, with larger LPC amplitudes to congruent than incongruent condition. This result is consistent with previous research [26,45], but differs from that found for the face task and conflict monitoring theory. LPC amplitude increases with the importance of the stimulus and with motivated attention [56]. We suggest that this may reflect evidence of a modality-specific processing order. Unlike faces, since semantic coherence is already involved in speech tasks at the N400 stage, the subsequent 500–650 ms positivity may reflect the facilitated allocation of evaluation weights and processing resources when acoustic impressions and trait meanings are congruent. We are cautious about this inference and need to test it in later studies. The interaction indicates that for low-attractive voices, consistency influenced the LPC, whereas it had no such effect for high-attractive voices. This pattern suggests that the consistency of trait words affected evaluative processing for low-attractive voices. This finding is consistent with the performance effects where only low-attractive voices are modulated by congruency. Additionally, it is also consistent with the observation of larger N1 amplitudes elicited by low-attractive voices, indicating that greater early attention was allocated to less attractive stimuli. For these perceptually ambiguous signals, the integration process appears to rely more heavily on contextual information. In the later 650–800 ms window, only the main effect of attractiveness persisted, with high-attractive voices eliciting larger amplitudes, possibly reflecting sustained positive appraisal or reward processing [45]. These effects support stereotype theory, as congruent positive traits reinforce the reward value of attractive voices, enhancing motivated attention, while low-level sequential processing of auditory cues may amplify the role of semantic context in early evaluation stages.

### 4.4. Integration of Results on Face and Voice Attractiveness

Overall, we found that personality traits exert both overlapping and distinct influences on the perception of facial and vocal attractiveness, our findings highlight the complex interaction between personality traits and attractiveness perceptions, which appear to be temporally and modality specific. Behaviorally, our results indicate that in both the face and voice domain, the congruency between personality traits and attractiveness categories significantly affects attractiveness judgments, supporting the “what is good is beautiful” stereotype where positive traits amplify perceived attractiveness across modalities [3,5]. In terms of ERP findings, we observed modality-specific differences in the cognitive processing of these influences. In face and voice tasks, early ERP components (P1/N1/N170 for faces; N1 for voices) were modulated only by attractiveness and not by congruency. This is consistent with the idea that the perceptual processing of attractiveness (visual/auditory) occurs before the integration of personality trait words in a semantic manner. It likely reflect initial sensory processing and attention allocation to the stimulus [72]. These early effects may be driven by low-level perceptual differences, with faces processed holistically and voices sequentially, influencing the timing of trait integration [2,32]. In line with this staging, the face task did not show an N400 congruency effect, suggesting that the visual salience of faces dominates early processing and delays, rather than eliminates, the semantic impact of trait information at this stage, and it shows the conflict is not processed at the semantic stage for faces. In contrast, the voice task exhibited an N400 congruency effect, consistent with relatively earlier access to semantic consistency for vocal signals.

Furthermore, the processing of personality trait information diverged temporally between the two modalities. In the face task, the impact of personality traits became apparent during the late-stage LPC, reflecting higher-level cognitive integration and evaluative processing. Conversely, in the voice task, this influence was evident as early as in the N400 component, indicating that semantic incongruency between personality traits and vocal attractiveness was detected and processed at an earlier stage of semantic integration. These differences highlight the complex interplay between perceptual cues and semantic information in shaping attractiveness judgments across different sensory modalities and underscore the need for further research to explore these interactions in more diverse populations and contexts. Future studies could further disentangle whether low-level perceptual factors, such as face configuration versus vocal prosody, or higher-level stereotype expectations drive these modality-specific effects.

### 4.5. Limitations

The present study has several limitations. Firstly, although in line with power calculations, the sample size was relatively small, and to reduce the complexity of the research, we only recruited female participants who only evaluated male faces and voice. Although the large effect sizes observed lend support to the findings, a larger and more diverse sample would enhance the stability of the experimental results and improve ecological validity. Of specially interest would be a fully factorial design with male and female participants and male and female stimuli. In addition, the 600 ms blank screen before responses may introduce (as we believe at most) minor motor preparation effects, future studies could use longer delays or response-locked analyses to further isolate cognitive signals. Furthermore, subsequent studies should more thoroughly investigate the degree to which alleged personality traits influence attractiveness. For example, including a neutral-attractiveness level in the stimulus set may allow a clearer comparison of how personality trait descriptors influence attractiveness ratings and how they interact with baseline perceptual attractiveness, thereby improving the separation between attractiveness baselines and trait-driven effects. Furthermore, although counterbalancing was carried out to rule out order effects such as fatigue or practice, there may be carry-over effects between tasks, which provide a caveat for the present results and could be ruled out in research with between group designs. In our study, a potential problem may lie in the asymmetric differences between attractive and unattractive stimuli. This makes it unclear whether the asymmetric congruency effects relate to the valence of the stimuli or to the asymmetric difficulty of classification. Further research should use more symmetrical stimuli to examine the influence of personality traits on attractiveness.

## 5. Conclusions

This study employed ERP technology and a Stroop-like paradigm to explore the impact of personality traits on the perception of faces and voices at different attractiveness levels. The results indicate at least partially modality-specific effects of alleged personality characteristics on attractiveness: in the face task, this influence appeared primarily in a late LPC stage, while in the voice task, it was observed in the N400 and early LPC stages. Moreover, negative personality traits enhanced judgment agreement in low-attractive conditions, especially in the voice task. Together, these findings suggest that the modulation of attractiveness by personality traits in social cognition is both temporally sequenced during cognitive processing stages and partially modality-dependent.

## Data Availability

The raw data supporting the conclusions of this article will be made available by the authors on request. The data are not publicly available due to privacy.
